# Resolution of Paediatric Cow's Milk Allergy Is Associated With Low Tfh Cell Frequencies and Elevated IL‐17 and IL‐22 Levels

**DOI:** 10.1111/cea.70388

**Published:** 2026-07-12

**Authors:** Guillaume Lezmi, Celine Dietrich, Marie‐Alexandra Alyanakian, Messaouda Attailia, Simon Fillatreau, Lucienne Chatenoud, Karine Adel‐Patient, Maria Leite‐de‐Moraes

**Affiliations:** ^1^ Team Immunity in Health and Disease Institut Necker Enfants Malades, Inserm UMR‐S1151, CNRS UMR‐S8253, Université Paris Cité Paris France; ^2^ Service de Pneumologie et Allergologie Pédiatriques Hôpital Necker‐Enfants Malades, AP‐HP Paris France; ^3^ Service d'Immunologie Biologique Hôpital Necker‐Enfants Malades, AP‐HP Paris France; ^4^ Team Normal and Pathological Lymphoid Differentiation Institut Necker Enfants Malades, Inserm UMR‐S1151, CNRS UMR‐S8253, Université Paris Cité Paris France; ^5^ Faculté de Médecine Université Paris Cité Paris France; ^6^ Laboratoire d'Immuno‐Allergie Alimentaire, SPI, Département Médicaments et Technologies pour la Santé (DMTS) Université Paris‐Saclay, CEA, INRAE Gif‐sur‐Yvette France

**Keywords:** children, cytokines, food allergy, T cells, tolerance

## Abstract

- Distinct immunological patterns are associated with clinical tolerance in paediatric IgE‐mediated cow's milk allergy.
- Circulating cytokines and T cells represent promising noninvasive biomarkers for CMA diagnosis and prognosis in infants.

**Abbreviations**
CM: cow's milk
CMA: IgE‐mediated cow's milk allergy
OFC: oral food challenge
Tfh: T follicular helper cells
Th2: CD4^+^ type 2 helper cells
Treg: regulatory T cells

To the Editor,

IgE‐mediated cow's milk (CM) allergy (CMA) is among the most prevalent food allergies in children. Affected individuals typically exhibit elevated levels of allergen‐specific IgE and an expansion of allergen‐specific CD4^+^ type 2 helper (Th2) lymphocytes compared with non‐allergic children [1]. Approximately 70% of children with confirmed CMA can tolerate baked CM and are only reactive to raw CM [1, 2]. Moreover, 70% of children will naturally outgrow their allergy and develop tolerance to raw CM before the age of 6 years [2, 3]. However, the immunological mechanisms underlying the acquisition of tolerance versus the persistence of allergy remain poorly understood.

We aimed to determine whether distinct T‐cell subsets and circulating cytokine profiles are associated with clinical tolerance to CM. We enrolled 58 children with confirmed IgE‐mediated CMA the day of an oral food challenge (OFC) performed with either baked or raw CM to assess their tolerance status. Diagnosis of CMA was established according to current clinical guidelines, based on a convincing history of immediate (< 2 h) allergic reactions (cutaneous, respiratory, gastrointestinal, or anaphylactic) following CM ingestion, together with evidence of IgE sensitisation (skin prick test ≥ 3 mm and/or CM‐specific IgE > 0.1 kU/L). At the time of the OFC, all children were asymptomatic without evidence of ongoing infection or acute inflammatory conditions. Blood samples were obtained just before the OFC challenge. Flow cytometry analyses were performed immediately, and plasma samples were stored for subsequent cytokine measurements.

Children were stratified into four groups based on the result of their OFC: baked milk–allergic (BMA; *n* = 9; 8 males; median age 7.5 years [7.0–8.5]), raw milk–allergic (RMA; *n* = 20; 11 males; median age 10.0 years [8.2–15.7]), baked milk–tolerant (BMT; *n* = 12; 4 males; median age 11.0 years [8.1–13.5]) and raw milk–tolerant (RMT; *n* = 17; 13 males; median age 10.0 years [7.0–12.5]). Children were classified according to their reactivity to baked and raw cow's milk, reflecting a putative continuum of disease resolution. BMA children represented the most severe phenotype of CMA. RMA children reacted to raw milk but tolerated baked milk, suggesting partial acquisition of tolerance. BMT children exhibited an intermediate phenotype, being either allergic only to raw milk or fully tolerant to both baked and raw milk. Finally, RMT children did not react to raw cow's milk and were considered to have achieved complete clinical tolerance. This classification was used to explore whether immune parameters evolved progressively along the spectrum from persistent allergy to natural resolution. Disease severity was hierarchically defined based on OFC responses (BMA > BMT > RMA > RMT).

Peripheral T‐cell cytokine profiling, performed as previously described [4], revealed no significant differences in the frequency of IL‐4, IL‐13 and IL‐17‐producing CD4+ T cells but an enrichment of IFN‐γ‐ and TNF‐α‐producing CD8^+^ T cells, as well as TNF‐α–producing CD4^+^ T cells across groups, with significantly lower frequencies observed in BMA compared with RMT (Figure 1A). In addition, the frequency of naïve CD4^+^ T cells was significantly lower in the BMA group than in the RMT group (Figure 1B). Conversely, regulatory T (Treg) cells were more frequent in BMA compared with RMT (Figure 1B). T follicular helper (Tfh) cells were more abundant in BMA than in BMT, RMA and RMT groups (Figure 1B). Correlation analyses demonstrated a positive association between Tfh and Treg frequencies (*r* = 0.311, *p* = 0.029), as well as between Tfh cells and CM‐specific IgE levels (*r* = 0.435, *p* = 0.005), suggesting coordinated regulation of humoral immunity and T‐cell responses in CMA [5].

**FIGURE 1:**
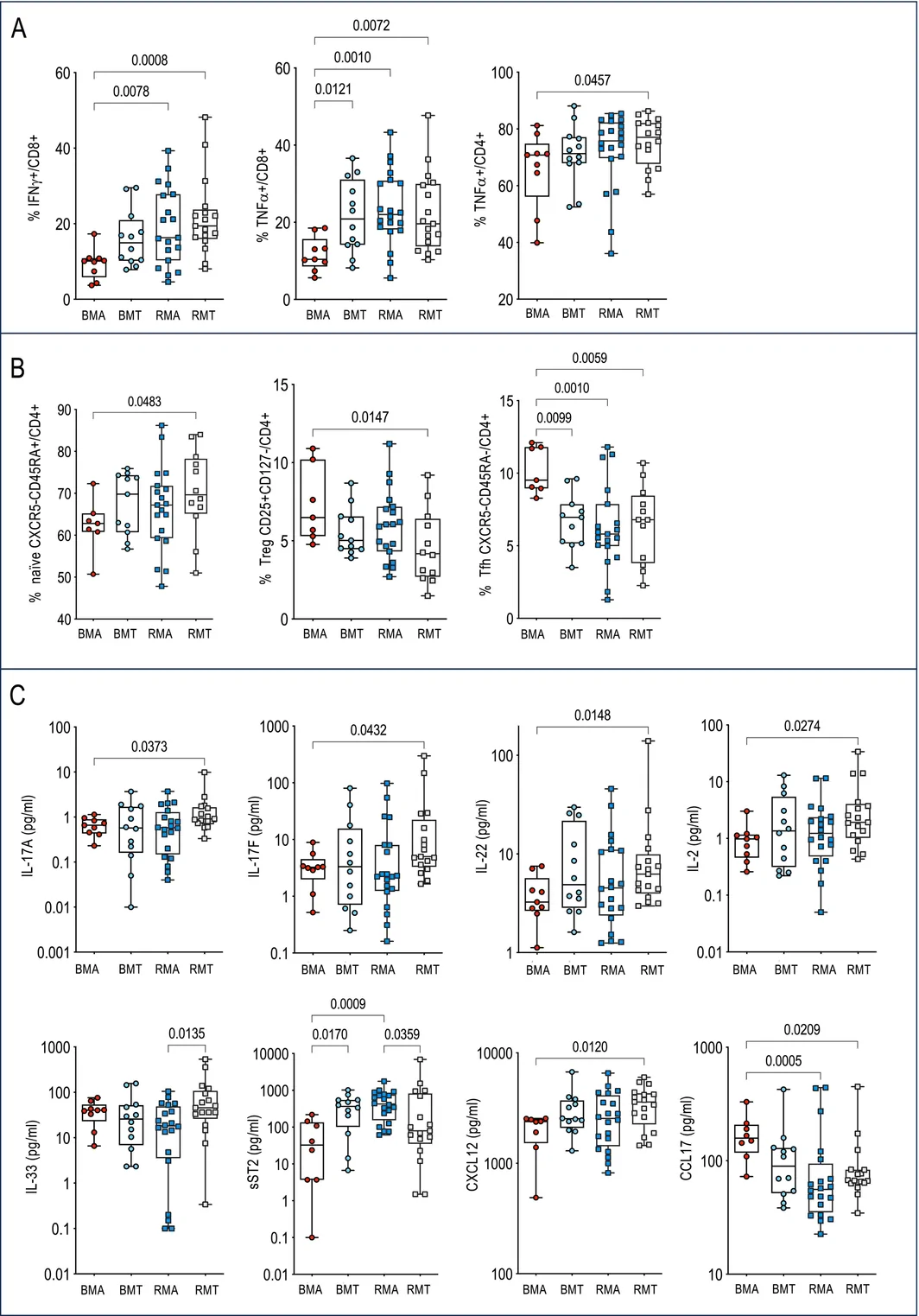
Blood cellular and humoral profiling in children with diagnosed CMA (Department of Paediatric Pulmonology and Allergy of Necker Hospital (Paris, France); NCT03776474). (A) Frequencies of IFNγ+ and TNFα+ among gated CD4+ T cells and TNFα+ and IFNγ+ among gated CD8+ after 6 h with 25 ng/mL phorbol 12‐myristate 13‐acetate (PMA) and 10^−6^ M ionomycin, in the presence of 10 ng/mL Brefeldin A. (B) Frequencies of naive (CXCR5− CD45RA+), Tfh (CXCR5+ CD45RA−), Treg (CD25 + CD127int) among ex vivo gated CD4+ T cells. (C) Plasma levels of IL‐17A, IL‐17F, IL‐22, IL‐2, IL‐33, sST2, CXCL12 and CCL17. Each point within the box plot represents one individual. Statistical differences were determined by Kruskal–Wallis test. Significant *p* values (*p* < 0.05) are indicated. BMA, baked milk‐allergic; BMT, baked milk tolerant; RMA, raw milk‐allergic; RMT, raw milk tolerant.

Plasma profiling of 49 cytokines and chemokines revealed distinct patterns associated with tolerance. Levels of type 17–associated cytokines IL‐17A, IL‐17F and IL‐22 were significantly lower in BMA relative to RMT (Figure 1C). IL‐2, a key cytokine for T‐cell proliferation and activation, was likewise reduced in BMA versus RMT (Figure 1C). IL‐33 and its decoy receptor soluble ST2 (sST2) were significantly lower in RMA as opposed to RMT, while sST2 levels were also diminished in BMA relative to BMT (Figure 1C). Among chemokines, CXCL12 levels were reduced, whereas CCL17 levels were elevated in BMA compared to RMT (Figure 1C). Notably, CCL17 levels were similarly lower in RMA when contrasted with BMA (Figure 1C). No significant differences were observed in the levels of IL‐10 and TSLP, similarly to the others 39 cytokines and chemokines tested [4].

Taken together, the acquisition of CM tolerance was associated with lower frequencies of Tfh and Treg cells and higher circulating levels of IL‐17A, IL‐17F and IL‐22. The lower frequency of Treg cells in tolerant individuals may appear counterintuitive, given their well‐established role in immune regulation and tolerance. However, previous studies have reported increased CM‐specific Treg cells in allergic individuals, possibly reflecting a compensatory response to ongoing inflammation [6]. Similarly, the higher frequency of Tfh cells in BMA, which are key drivers of B‐cell activation and IgE production, further supports their contribution to pathogenic humoral responses.

The functional roles of IL‐17A, IL‐17F and IL‐22 in allergic disease remain incompletely defined. IL‐17 is generally considered a pro‐inflammatory cytokine that contributes to host defence but may also promote pathogenic inflammation in conditions such as toxic epidermal necrolysis and allergic contact dermatitis. In contrast, IL‐22 appears to exert tissue‐protective effects and contributes to epithelial barrier integrity, particularly in early stages of diseases such as asthma and atopic dermatitis [7].

The pro‐Th2 effects of IL‐33 have already been described in allergic contexts [8]. In the present study, we observed that IL‐33 levels were higher in RMT than in RMA. However, a similar trend was observed for sST2 levels, suggesting that this decoy receptor may modulate the pro‐Th2 effects of IL‐33. CXCL12 is a chemokine involved in lymphocyte recruitment and in multiple pathological processes, including fibrosis. RMT exhibited elevated levels of this chemokine; however, its role in food allergy remains unclear. In contrast, the reduced levels of CCL17 observed in RMT‐tolerant individuals are consistent with its established role in Th2 cell recruitment and allergic inflammation [9].

This study has limitations, notably the relatively small sample size. Nevertheless, all participants were carefully phenotyped using OFC, the gold standard for diagnosing food allergy and assessing tolerance.

In conclusion, these findings identify distinct T cell and cytokine signatures associated with CM tolerance in children. In particular, Tfh and Treg cells, along with circulating IL‐17A, IL‐17F and IL‐22, could emerge as potential biomarkers of disease trajectory. These results underscore the complexity and heterogeneity of CMA in children and support the need for integrated immunological profiling in well‐characterised clinical cohorts. Longitudinal studies in larger populations will be essential to validate these biomarkers and to determine their utility in predicting the development of tolerance and response to immunotherapeutic interventions.

## Author Contributions

G.L., K.A.‐P. and M.L.M.: designed the research. C.D. and M.A.: performed the research. G.L., M.‐A.A. and L.C. were responsible for patient recruitment or establishing the patient clinical database. G.L., S.F., K.A.‐P. and M.L.M.: analysed the data. G.L., S.F., L.C., K.A.‐P. and M.L.M.: wrote the manuscript.

## Funding

This work was supported by the Inserm, the CNRS and the Université Paris Cité, Paris, France.

## Disclosure

Open‐Access Repository Accession: Zenodo open‐access repository and can be accessed at the following link: https://zenodo.org/records/20702922.

## Ethics Statement

This study was approved by the institutional review board of Necker Hospital (NCT03776474).

## Consent

Written informed consent was obtained from the parents or legal guardians of all participating infants prior to inclusion in the study.

## Conflicts of Interest

The authors declare no conflicts of interest.

## Data Availability

The data that support the findings of this study are available from the corresponding author upon reasonable request.
